# Measuring the Immediate Effects of High-Intensity Functional Training on Motor, Cognitive and Physiological Parameters in Well-Trained Adults

**DOI:** 10.3390/s23083937

**Published:** 2023-04-12

**Authors:** Luca Molinaro, Juri Taborri, Denis Pauletto, Valentina Guerra, Damiano Molinaro, Giovanni Sicari, Antonello Regina, Enrico Guerra, Stefano Rossi

**Affiliations:** 1 Department of Economics, Engineering, Society and Business Organization (DEIM), University of Tuscia, 01100 Viterbo, VT, Italy; 2 ELAV, 06012 Città di Castello, PG, Italy

**Keywords:** HIFT, well-trained, immediate effects, physical activity, body stability, cognitive, jumping

## Abstract

The importance of physical activity has been widely demonstrated both in clinics and in sports. One of the new frontier training programs is high-intensity functional training (HIFT). The immediate effects of HIFT on the psychomotor and cognitive performance of well-trained people are still not clear. This paper aims to evaluate the immediate effects induced by HIFT on blood lactate levels, physical performance in terms of body stability and jump ability, and cognitive performance in terms of reaction time. Nineteen well-trained participants were enrolled in the experimental studies and asked to execute six repetitions of a circuit training. Data were gathered both in a pre-training session and after each one of the circuit repetitions. An immediate significant increase with respect to the baseline was observed during the first repetition, with a further increase after the third one. No effects on jump ability were found, whereas a deterioration in body stability was found. Positive immediate effects on cognitive performance in terms of accuracy and speed in task execution were assessed. The findings can be exploited by trainers during coaching to optimize the design of training programs.

## 1. Introduction

Physical activity leads to a series of benefits for the health status of subjects [1]. In fact, major associations for disease prevention recommend the regular practice of moderate-intensity activity in order to prevent the occurrence of disorders [2].

Among exercise programs, high-intensity interval training (HIIT) has been recognized as a valuable protocol and become very popular in the last decade [3]. HIIT is characterized by repeated short-to-long bouts of intensive exercises [4]. Due to its potentiality, HIIT has been already used both in clinical settings, i.e., for the prevention of and/or to treat cardiac disorders [3], and in sports, i.e., to improve the performance of elite athletes [5]. As an example, HIIT positively affected the maximum oxygen uptake and anaerobic power in a long-term analysis when testing non-sedentary young adults [6]. Compared to continuous training, HIIT showed its potential to increase cognitive performance in young healthy adults, especially regarding reaction times [7]. Bahdur et al. proposed an experimental study to evaluate the immediate effects of HIIT on physical and cognitive performance [8]. The authors collected data from 44 recreationally active university students and found that the exercise program positively impacted cognitive function, whereas the improvement in reaction times and the decrement in physical performance were not significant. A single bout of HIIT was also demonstrated to lead to an improvement in the brain-derived neurotrophic factor and vascular endothelial growth factor of healthy young men [9]. However, the immediate effects of HIIT are often contradictory, especially in sports [10].

Despite the popularity achieved by HIIT, the new frontier in training modality is represented by high-intensity functional training (HIFT). HIFT mainly emphasizes functional and multi-joint movements that elicit greater muscle recruitment [11]. Focusing on HIFT, Wilke proposed an experimental protocol to evaluate the effects of HIFT on cognitive performance in 35 healthy individuals. The author discovered the potential of HIFT to increase working memory [12]. The fatigue induced by two sessions of HIFT has been assessed in [13]; the study revealed that recovery in terms of neuromuscular factors, such as velocity and strength in task execution, is different with respect to metabolic recovery, evaluated via blood lactate concentration. The acute effect of HIFT on anaerobic performance was assessed when acquiring data from a cohort of kickboxers at different times of the day. The results showed that HIFT had better effects in the evening rather than at other parts of the day on vertical jump and peak power [14].

From the literature review, few studies have been published regarding the immediate effects of HIFT on physical and cognitive performance. Thus, a full understanding of the immediate effects of HIFT remains elusive, especially when referring to well-trained adults. This knowledge could provide coaches with precise guidelines to follow in the implementation of specific training programs. To the best of the authors’ knowledge, no studies have been conducted on well-trained young adults in the evaluation of the immediate effects induced by HIFT. Contextually, no sensor-based protocols have been implemented to assess such effects; for instance, the effects on stability have not been considered, despite body stability being fundamental in several sports [15]. In addition, no studies have been conducted to evaluate the trend of effects induced by HIFT by measuring physiological parameters after each repetition of an exercise. This approach would also allow one to estimate the correct number of repetitions to be considered during the implementation of a HIFT protocol. From this perspective, the aim of this paper is to evaluate the immediate effects of a high-intensity functional training program performed by well-trained young adults. A sensor-based procedure was implemented to gather information in terms of (i) physiological parameters, i.e., blood lactate; (ii) physical performance by considering jump and stability tests; and (iii) cognitive performance in terms of reaction times. Our hypothesis is that HIFT, different to HIIT [10], could be more useful in sports by avoiding the negative immediate effects induced by cognitive and physical fatigue when considering well-trained subjects. Furthermore, we believe that it is important to understand the number of maximum repetitions of an exercise to avoid negative effects. The results of this paper could be exploited to improve the quality of training programs in order to enhance athlete performance.

## 2. Materials and Methods

### 2.1. Participants

Nineteen well-trained male adults were involved in the study. As inclusion criteria, all enrolled participants had practiced HIFT for at least three years at least four times per week. Participants who had suffered injuries and/or undergone orthopedical surgery were excluded. All participants were informed of the rationale of the study and written informed consent was obtained, according to the ethical standards outlined in the 1964 Declaration of Helsinki. All the procedures were conducted in the ELAV training and functional assessment center.

#### BIA Analysis

Body composition parameters were acquired. A bioelectrical analyzer (BIA 101 BIVA PRO, Akern, Florence, Italy) at a single frequency of 50 kHz was used. After cleaning the skin with isotropic alcohol, four low intrinsic impedance adhesive electrodes (Biatrodes Akern Srl, Florence, Italy) were placed on the participants’ right hands and right feet, following the standard protocol [16]. Phase angle (Pha), body resistance (R) and body reactance (X_c_) values were measured directly and automatically for the whole body. The BIA measurements were all performed by the same trained investigator to avoid inter-rater errors. The body composition values, which are presented in Table 1, were comparable to the ones reported by Campa et al. when referring to athletes [17].

### 2.2. Experimental Protocol and Setup

**Figure 1:**
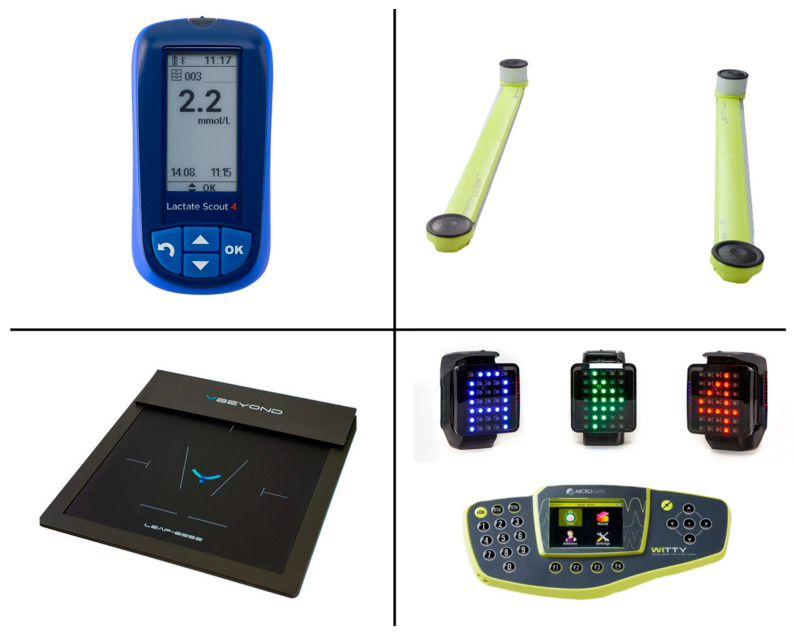
From top left: Lactate Scout 4, Optojump Next, Beyond Pressure and WittySEM photocells.

The experimental protocol consisted of four functional tests and the execution of a high intensity functional training (HIFT) circuit. The four functional tests included the evaluation of lactate levels using the lactacidimeter Lactate Scout 4 (SensLab GmbH, Leipzig, Germany), analysis of the oscillation of the COP in monopodalic balance for both legs using the Beyond Pressure 60 × 50 platform (Motustech, Rome, Italy), evaluation of jump height during repeated jumps using the Optojump Next system (Microgate, Bozen, Italy) and analysis of responses during a cognitive task using the WittySEM photocell system (Microgate, Bozen, Italy). The four devices are shown in Figure 1.

#### 2.2.1. Lactate Levels

Lactate levels [La^−^]_b_ were acquired with the Lactate Scout 4 (SensLab GmbH, Leipzig, Germany), a capillary blood lactate detector that uses an enzymatic-amperometric method. The lactate in the sample is oxidized by the enzyme lactate oxidase and, in the course of this redox reaction, electrons are transferred via an additional mediator from the enzyme to a functional electrode. The resulting current corresponds to the lactate concentration of the sample. Lactate Scout 4 requires a 0.5 µL blood sample and has a sample analysis time of 15 s. The test strips fill by capillary action directly from the sample site and each lot of test strips has a unique calibration code. Previous studies have analyzed the accuracy and reliability of this device and it is now widely used in sports practice [18,19]. Lactate level [La^−^]_b_ analysis was performed by an experienced operator collecting blood samples from the left earlobe. Before each acquisition, the lobe surface was disinfected and thoroughly dried. To avoid any unclean blood sampling, the first drop was eliminated. The sampling was performed within 30 s after the end of each circuit in order to compare the [La^−^]_b_ after all the circuit executions at the same time. It is worth noting that such an approach did not allow for the construction of a lactate curve since the maximum peak generally occurred after the first minute; however, we sought to evaluate the level of [La^−^]_b_ at a certain interval after the execution of each task, regardless of the peak.

#### 2.2.2. Repeated Jumps

**Figure 2:**
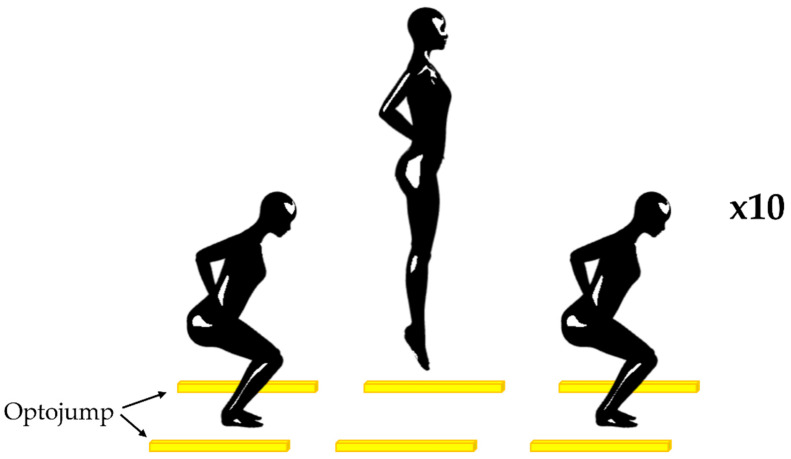
Repeated Jumps: positioning of the subject and optoelectronic bars.

Repeated Jumps (RJ) were assessed with the Optojump Next system (Microgate, Bozen, Italy), which transmits-receives 1 m long bars positioned above the ground. Each bar is equipped with 96 LED diodes. Data were recorded using Optojump Next Version 1.12.23 software (Microgate, Bozen, Italy). Through this device, it is possible to record the flight and contact times of each foot with a resolution of 1 ms. The sampling frequency was set at 1000 Hz. The Optojump Next system is generally used in the analysis of the flight and contact times of jump gestures [20,21,22]. In the RJ test, subjects were asked to perform 10 maximal jumps, starting from a standing position, with hands on hips, positioning themselves centrally between the two optoelectronic bars, which were placed at a distance of 2.5 m (Figure 2). They were instructed to keep their hands in contact with their body for the whole task and were asked to jump as high as possible, minimizing the ground contact time [23].

#### 2.2.3. Monopodalic Balance

**Figure 3:**
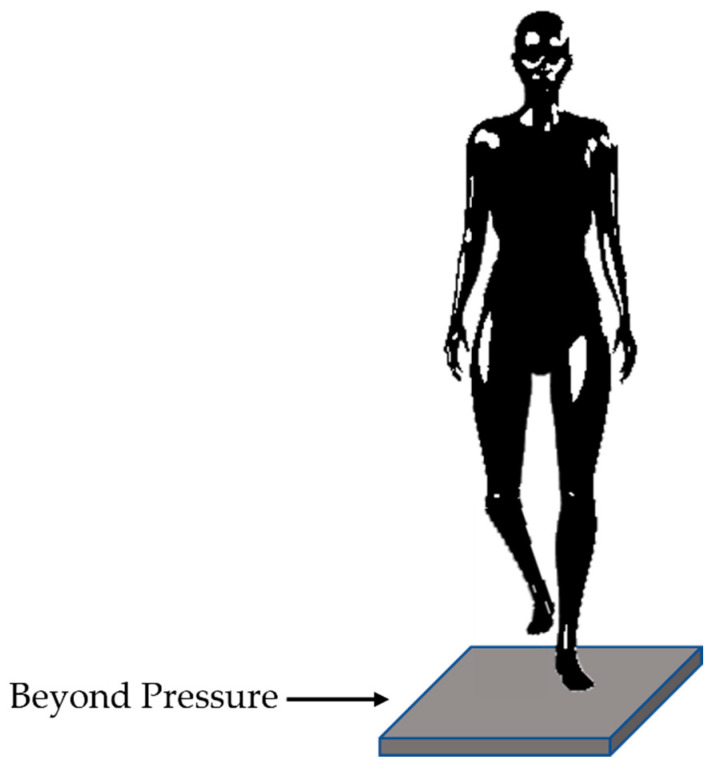
One-leg standing balance test: positioning of the subject and platform.

The evaluation of the balance test was conducted using the Beyond Pressure 60 × 50 platform designed by Motustech (Rome, Italy). The platform is composed of 3000 resistive sensors arranged according to 50 rows and 60 columns, with a total area of 598 × 518 mm^2^. The sensitive area of each sensor is 1 × 1 cm^2^. The full scale of the sensors is 150 N/cm^2^, with an acquisition frequency of up to 400 Hz. The matrix is connected to the PC via a USB cable and interfaces with the Beyond Framework Software (Motustech, Guidonia (RM), Italy, version 1.1.0.3). The matrix is covered with a conductive synthetic rubber material in socaprene. The reliability and repeatability of the Beyond Pressure system have already been analyzed by the same authors in a previous study [24]. A one-leg standing balance (OLSB) test was performed [25]: more specifically, subjects were instructed to stand with their arms relaxed at their sides and one limb lifted off the ground by flexing the knee and hip, holding the position for 30 s (Figure 3). The test was performed with open eyes looking at a fixed point on the wall at a distance of 5 m. The subjects were asked to mount the pressure platform without shoes and perform the OLSB test with the right and left limbs, sequentially. Data were gathered from the platform only during the OLSB tests.

#### 2.2.4. Cognitive Task

**Figure 4:**
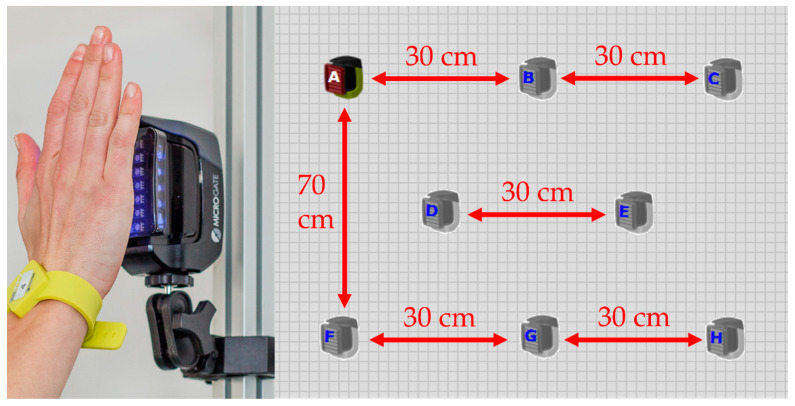
Reactivity test: positioning of the Witty SEM system.

The acquisition of the cognitive tasks was performed through the WittySEM photocell system (Microgate, Bozen, Italy), which has eight photocells, each with an optical proximity sensor and a 7 × 5 LED matrix that can display different colors, arrows in several directions, numbers, letters, and other symbols. The proximity sensor allows one to set the distance between the subject and each photocell by approaching the hand without touching it. In this case, the distance was set to 10 cm. A tablet (WittyTIMER) manages all the photocells via radio communication in real time for the acquisition of events. This system is currently widely used in sports, especially for the evaluation of tasks, such as change of direction, and the training of athletes [26]. The cognitive task consisted of a reactivity test (RT) where the subject had to distinguish the LED target based on the color. The LED matrices were positioned as shown in Figure 4 by attaching them to a metal structure using the clamps supplied by the company. The subject was positioned at a distance of 60 cm from the structure in a monopodalic support using the dominant limb. The subject was asked to approach the hand as quickly as possible to the only LED matrix, where a green rectangle was shown in order to “turn off” the LED. The other LED matrices showed rectangles with different colors. During the execution of the test, the subject could use both upper limbs to turn off the LED matrices. A new sequence of symbols was shown on the LED matrices after 300 ms. The sequence of the target was random. The test had a total duration of 30 s. The RT test is generally used as a method to evaluate the time between the stimulus and the response of a subject [27]. Figure 4 shows a schematic of the WittySEM positioning.

Before starting the data acquisition, a familiarization phase was performed in order to make subjects aware of the required experimental tasks and avoid bias in the results due to learning processing, especially concerning the cognitive tests. Tests were performed in the sequence just described with a 30 s pause; the whole sequence was completed in approximately 4 min.

After a 5 min warm-up with free movement exercises (hopping, skipping and running backwards), tests were performed before starting the HIFT to create reference values (PRE) and at the end of each HIFT completion (POSTn), where n indicates the number of repetitions. Specifically, the HIFT was performed six times, leading to six evaluations of the POST parameters. The pause between two repetitions of a HIFT circuit was approximately 5 min for the execution of the functional tests.

#### 2.2.5. High-Intensity Functional Training Circuit

**Figure 5:**
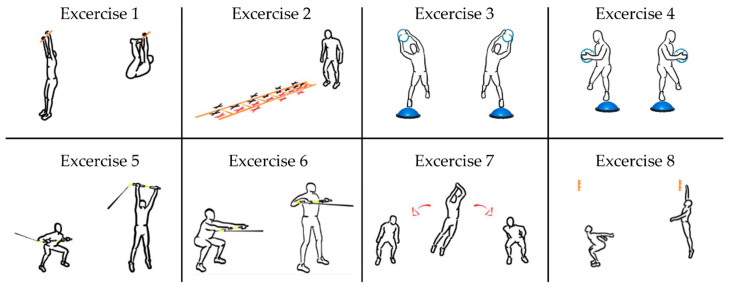
High-intensity functional training circuit.

The training circuit consisted of eight exercises involving the whole body performed in sequence, as shown in Figure 5. A workstation was dedicated to each exercise. The duration of each exercise was 30 s, with a 30 s break between each exercise. Participants were asked to exert maximum effort during the execution of the exercises and attempt to carry out the greatest number of repetitions while maintaining correct execution. For this scope, an operator provided verbal encouragement during each exercise to ensure that the participants performed the exercise at high intensity. In the following section, the details of each exercise are reported:

- In the first exercise, the subject hung from a bar using their hands and, for each repetition, had to bring their feet towards the bar by pulling their legs up [28].
- The second exercise was an agility ladder drill [29] in which the subject performed a low skip with his legs using the ladder placed on the ground, alternating between right and left supports.
- The third exercise involved a series of lunges on one leg, alternating right and left, on a bosu while the subject held a 3 kg medicine ball with arms extended above the head and then performing a side bend using the trunk [30].
- The setup of the fourth exercise was similar to that of the third but, in this case, the subject twisted their trunk while holding the medicine ball, with their arms folded in front of their chest [30].
- The fifth exercise involved the use of a bar, with one end connected by a rubber band attached to the wall. The subject took the bar with a shoulder-width grip using two hands and pre-tensioned the elastic; at this point, starting from a squatted position with the bar in front of the chest and the wall behind them, the subject went into an upright position, rising onto their toes, carrying the bar with their arms above the head and then returning to the starting position [31].
- The sixth exercise was similar to the fifth but, in this case, the subject started from a squatting position with the wall in front of them, keeping their arms extended and the elastic pre-stretched. At this point, the subject stood up into an upright position by pulling the bar to their chest and then returned to the starting position [31].
- The seventh exercise consisted of a series of lateral leaps with a minimum distance of 2 m, indicated by two stickers placed on the ground that were clearly visible to the subject [32].
- The eighth exercise consisted of a series of vertical bipedal jumps in which the subject had to try to touch the highest possible point with one hand [33].

### 2.3. Data Analysis

For each subject, the outputs of the four sensor systems were individually analyzed according to the steps reported in the following sections.

#### 2.3.1. Lactate Level

After taking the blood sample on the test strip, it was inserted into the device until the measuring chamber was filled. After approximately 15 s, the result of the [La^−^]_b_ [mmol/L] appeared on the device display and was then recorded.

#### 2.3.2. Monopodalic Balance

Body stability was analyzed, considering the center of pressure (CoP) provided by the platform. The coordinates of the CoP measured with the pressure matrix with *N* total sensors were obtained by the following equation, as indicated in [34]:

(1) $$\left[\begin{array}{c} {CoP}_{x}\\ {CoP}_{y} \end{array}\right]=\frac{1}{\sum_{k}^{N}P_{k}*A_{k}}\left[\begin{array}{c} \sum_{k}^{N}P_{k}*A_{k}*x_{k}\\ \sum_{k}^{N}P_{k}*A_{k}*y_{k} \end{array}\right]$$

where (*x_k_*, *y_k_*) are the coordinates of the position of the *k*th sensor; *P_k_* is the pressure at the *k*th sensor and *A_k_* is the area of the *k*th sensor.

The data were acquired at a frequency of 25 Hz. The CoPs of both the right and left stances were analyzed. Finally, we computed stability indices that are typically considered in posturographic analyses by following the equations reported in [35], particularly the path length (PL) and ellipse area (EA):

- Path Length (PL), which is the total length of the CoP path in the plane;
- Ellipse Area (EA), which is the minimum area of the bivariate confidence ellipse that contains at least 95% of the path points.

#### 2.3.3. Repeated Jumps

As regards the RJ, the instants of time in which the subject touched and detached the ground were measured using the Optojump Next system. This system identifies these instants through the on-off status change of the LEDs of the optoelectronic bar. From the time-of-flight values, the system provides an estimation of the jump height (JH) using the following formula:

(2) $$JH=\frac{T_{v}^{2}*g}{8}$$

where *T_v_* is the time of flight and *g* is the gravity acceleration.

A total of eight jumps were analyzed, discarding the first and the last jumps. At this point, the average of the eight jumps (JH_A_) and the highest jump (JH_M_) were calculated.

#### 2.3.4. Cognitive Task

As regards the RT, the number of correct answers (CA) that the subject was able to provide in 30 s, the average response time (ART) and the number of errors committed (NE) were analyzed.

### 2.4. Statistical Analysis

Data were first tested for normality with the Shapiro Wilk test. The means and standard deviations across subjects were computed for each parameter. [La^−^]_b_, EA and PL were tested by one-way ANOVAs, whereas one-way repeated measure ANOVAs were applied to JH_A_, JH_M_ and ART. ANOVA was used in order to identify the presence of statistical differences among the experimental sessions, considering both the PRE and the POST sessions, in order to identify the effects induced by HIFT along the time execution. For all ANOVA tests, Bonferroni post hoc multiple comparison tests were used to determine differences among means when the ANOVA test was significant. As regards the parameters CA and NE, non-parametric Kruskal-Wallis tests with independent k-samples were performed and Dunn post hoc multiple comparison tests were used to determine differences between PRE and POST sessions. Statistical significance was set at *p* < 0.05 for all the performed tests. The SPSS 17.0 software application for Windows was used for statistical analysis. The statistical power of the analysis was computed with G*Power software [36]. The outcomes of the power statistical analysis showed a mean power value of approximately 85%, with a medium effect size (0.5) [37].

## 3. Results

### 3.1. Lactate Level

The mean values and standard deviations of [La^−^]_b_ for each experimental session are reported in Table 2.

Analyzing the results of [La^−^]_b_, statistical differences emerged between PRE and all POST (*p*-value < 0.001); between POST1-PRE (*p*-value < 0.001), POST1-POST3 (*p*-value = 0.001), POST1-POST4 (*p*-value = 0.001), POST1-POST5 (*p*-value = 0.005) and POST1-POST6 (*p*-value = 0.008); and between POST2-PRE (*p*-value < 0.001), POST2-POST3 (*p*-value = 0.001), POST2-POST4 (*p*-value = 0.001), POST2-POST5 (*p*-value = 0.005) and POST2-POST6 (*p*-value = 0.008), while no differences emerged between POST3, POST4, POST5 and POST6. In particular, the lowest value was registered in the PRE. The values increased from PRE to POST3 and slightly decreased from POST3 to POST6.

### 3.2. Monopodalic Balance

The mean values and standard deviations of EA and PL for both the right and left sides for each experimental session are reported in Table 3.

By analyzing the results, significant differences only emerged between PRE and POST1 for the parameters EA_DX_ and EA_SX_, with *p*-values 0.025 and 0.049, respectively. In other cases, for EA_DX_, *p*-values ranged between 0.129 and 1 and for EA_SX_ *p-*, values ranged between 0.136 and 1. In particular, an increase in the CoP oscillation area was evident between the PRE and POST1 sessions. There were no significant differences between sessions for PL_DX_ and PL_SX_. For PL_DX_, *p-values* were always 1 and for PL_SX_, *p*-values ranged between 0.128 and 1.

### 3.3. Repeated Jumps

The mean values and standard deviations of JH_A_ and JH_M_ for each experimental session are reported in Table 4.

Analyzing the results, no significant differences emerged between the sessions, both for JH_A_ and JH_M_. For both parameters across all sessions, the *p*-value was always equal to 1.

### 3.4. Cognitive Task

**Figure 6:**
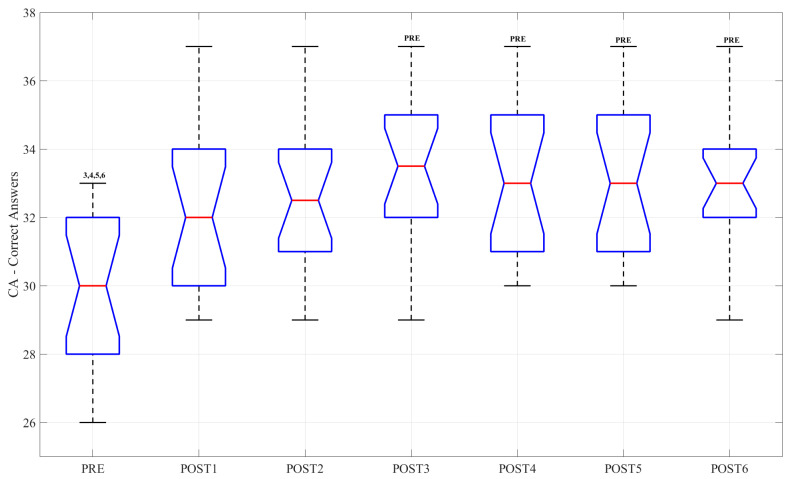
Boxplot related to CA. The central red mark indicates the median and the edges of the box are the 25th and 75th percentiles. Text in the upper section indicates differences between sessions.

**Figure 7:**
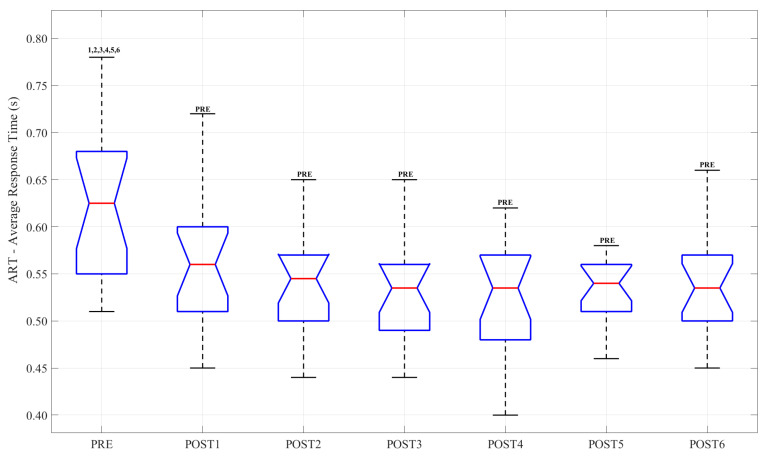
Boxplot related to ART. The central red mark indicates the median and the edges of the box are the 25th and 75th percentiles. Text in the upper section indicates differences between sessions.

Boxplots related to CA, ART and NE for each experimental session are reported in Figure 6 and Figure 7, respectively. In each graph, the significant differences are highlighted. From the analysis of the results, statistical differences emerged for the CA parameter between the PRE session and the POST3, POST4, POST5 and POST6 sessions (*p*-value = 0.007, *p*-value = 0.017, *p*-value = 0.012, *p*-value = 0.020, respectively). Concerning ART, significant differences emerged between PRE and all post sessions; specifically, POST3-PRE (*p*-value < 0.001), POST4-PRE (*p*-value < 0.001), POST5-PRE (*p*-value < 0.001) and POST6-PRE (*p*-value = 0.01). There were no differences between post sessions.

As far as NE is concerned, no significant differences emerged between any session. In particular, in all the sessions, more than two errors were not recorded and more than 75% of the values were equal to zero errors.

## 4. Discussion

We evaluated the effects induced by high-intensity functional training on physiological, physical and cognitive parameters by testing well-trained young adults. Tests were conducted by comparing the values of these parameters gathered in a pre-training session and after each of six repetitions of the HIFT.

By analyzing the results related to the physiological parameters, we found a significant increase in the level of lactate starting from the first post-exercise session, and a further increase after the third one. Compared to other studies [4,38,39], the slightly high values in the PRE session can be ascribed to the presence of the warm-up session. The level of lactate after each HIFT repetition showed lower values than the ones reported in [38]; this may be due to the methodology used to acquire the level of lactate. Specifically, we gathered values immediately after the end of each HIFT repetition and not in correspondence with the maximum peak since this was not the objective of the present study. Furthermore, the difference in lactate levels could be due to the duration of the exercises and the different recovery times, as highlighted by Warr-di Piero et al. [40]. In fact, lower lactate values emerged in the protocols with shorter times compared to the lengthier ones.

The trend that emerged for lactate values can be also associated with the significant difference found when observing cognitive performance. In fact, the metabolites resulting from exercise, such as blood lactate, have been demonstrated to play a fundamental role in improving cognitive performance [41]. In fact, lactate derived from the skeletal muscles is metabolized by neurons, with the direct consequence of enhancing executive functioning. Such consideration is justified by the ability of the central nervous system to improve the production of lactate in the substitution of low blood glucose resulting from aerobic exercise [42]. Similar to HIIT [8], high-intensity functional training leads to an increase in cognitive ability. More specifically, HIFT leads to an improvement in execution speed in terms of reaction times without compromising task accuracy (CA). The cognitive performance improvement after 8 min of HIFT was also proven when testing amatorial soccer players [43]. In addition, the absence of significant differences concerning the number of errors is in line with the results reported in [44]. Furthermore, we can conclude that HIFT is an effective method that causes acute responses, which lead to the enhancement of several aspects of cognitive performance, in addition to working memory as reported in [12]. Differently from other available studies, we evaluated how many repetitions of HIFT are needed to elicit such cognitive improvement. In fact, by considering the trend of the statistical results, we can speculate that accuracy in the execution of cognitive tasks does not vary after the third repetition, suggesting that the necessity of six repetitions is an overestimation in regard to planning training programs. This finding could be used as a starting point to solve one of the most unanswered questions when designing training, i.e., the ideal number of circuit repetitions; in fact, Jacob et al. found a strong connection between cognitive performance and the duration of an exercise program [4].

By moving to the physical parameters, we can affirm that the applied HIFT did not lead to a deterioration in physical performance when performing jump tasks due to the absence of significant differences, whereas a slight improvement of the average and maximum height of the performed jump was found. This result is in contrast with the literature, in which increases in lactate levels are associated with a decline in power outputs at the lower limb level when performing jumps due to an accumulation of positive hydrogen ions [44]. However, we can affirm that, even if associated with significant differences along the repetitions, blood lactate levels maintained low values, with an almost total absence of fatigue. In addition, the slight improvement in maximum height is in line with the ones reported in [14], where positive effects on jump ability due to HIFT were found in kickboxers. Thus, we can speculate that well-trained young adults, even if they do not participate in official competition as professional athletes, show similar behavior. Generally, the combination of results between cognitive and jump performance allows us to confirm that well-trained young adults are able to maintain their psychomotor ability during high-intensity functional training. This characteristic is one of the features that permits one to differentiate such a population from sedentary people, as reported in [43].

Contrary to jump ability, a significant difference was found when considering stability parameters in static balance tasks. In particular, increments in ellipse area considering both the right and left sides were shown. Thus, we can affirm that the first HIFT repetition causes an immediate effect on body stability. After the first repetition, an improvement in body stability can be observed. This outcome is in line with the results reported in [45]. In fact, the authors found that the effects of exercise on stability were short-lasting and, in general, were not liable to seriously threaten body equilibrium. This finding is of some interest since postural stability is often neglected among HIFT effects, despite being a fundamental motor ability in several sports.

We conclude that high-intensity functional training is associated with positive immediate effects on cognitive performance and that the duration of training programs is a parameter to take into account to avoid psychomotor deterioration. In addition, considering the almost total absence of significant negative effects on physical performance, it is not contraindicated to add high-intensity functional training into sports training programs, especially in sports in which the enhancement of cognitive skills is required, confirming our starting hypothesis. However, it is clear that a number of maximum repetitions should be set to avoid negative effects.

For completeness, this study reports some limitations. The cohort of subjects only included male well-trained adults; thus, gender effects were not considered. In addition, for a complete evaluation of professional sport, different types of athletes should be tested. Future developments will aim to test different types of subjects and training to obtain a wider perspective on the effects of HIFT.

## 5. Conclusions

Several previous studies affirmed that high-intensity training is a valuable training program for sports. Focusing on sports, the immediate effects induced by high-intensity functional training are still not clear in terms of cognitive and physical performance. Our results show that the intrinsic improvement of blood lactate levels is directly associated with an enhancement of cognitive performance in terms of both speed and accuracy in task execution. Our results also suggest that training duration and the number of circuit repetitions should be considered when focusing on the improvement of cognitive abilities. With the exception of an initial deterioration in body stability, no further negative effects on physical performance were found. These findings can be useful both for coaching and for designing appropriate training programs.

## Figures and Tables

**Table 1:** Participants’ details.

N = 19	Mean ± SD
Age (year)	28 ± 6
Height (cm)	177.3 ± 5.9
Weight (kg)	75.7 ± 7.5
Years of training (n)	11.2 ± 3.9
Body Resistance (Ω)	434.9 ± 40.6
Body Reactance (Ω)	57.9 ± 5.7
Phase angle (°)	7.5 ± 0.5

**Table 2:** Mean values (standard deviations) for [La^−^]_b_ obtained during all repetitions of the HIFT circuit. Superscripts represent statistical differences.

Sessions	Lactate Level ([La^−^]_b_) [mmol/L]
PRE	2.5 (0.9) ^POST1, POST2, POST3, POST4, POST5, POST6^
POST1	5.4 (1.2) ^PRE, POST3, POST5, POST6^
POST2	5.9 (1.3) ^PRE, POST3, POST5, POST6^
POST3	8.2 (2.2) ^PRE, POST1, POST2^
POST4	8.0 (2.1) ^PRE, POST1, POST2^
POST5	7.8 (2.7) ^PRE, POST1, POST2^
POST6	7.7 (2.4) ^PRE, POST1, POST2^

**Table 3:** Mean values (standard deviations) for EA_DX_, PL_DX_, EA_SX_ and PL_SX_ obtained during all repetitions of the HIFT circuit. Superscripts represent statistical differences.

Scheme 2.	EA_DX_ [mm^2^]	PL_DX_ [mm]	EA_SX_ [mm^2^]	PL_SX_ [mm]
PRE	1.5 (0.8) ^POST1^	25.9 (6.8)	1.5 (0.8) ^POST1^	24.9 (7.0)
POST1	2.7 (1.5) ^PRE^	24.7 (6.7)	2.7 (1.4) ^PRE^	27.1 (8.4)
POST2	1.9 (0.9)	26.1 (6.8)	2.4 (1.4)	27.6 (6.1)
POST3	1.9 (1.1)	24.4 (5.3)	2.3 (1.2)	26.1 (5.6)
POST4	2.1 (0.9)	26.5 (5.3)	2.3 (1.5)	23.9 (6.1)
POST5	2.1 (1.3)	23.3 (6.2)	2.1 (1.1)	21.8 (4.4)
POST6	1.7 (0.6)	24.3 (5.8)	2.0 (0.8)	24.5 (6.1)

**Table 4:** Mean values (standard deviations) for JH_A_ and JH_M_ obtained during all repetitions of the HIFT circuit. Superscripts represent statistical differences.

Sessions	JH_A_ [cm]	JH_M_ [cm]
PRE	31.3 (4.1)	33.9 (4.6)
POST1	32.5 (4.7)	34.9 (4.7)
POST2	33.5 (5.1)	36.3 (5.0)
POST3	32.5 (4.7)	34.5 (4.9)
POST4	32.7 (5.3)	35.1 (5.4)
POST5	33.6 (4.7)	36.0 (4.8)
POST6	32.9 (5.2)	35.5 (5.8)

## Data Availability

For data availability, please refer to the corresponding authors.
